# Factors associated with vaccination coverage in children up to 15 months old, born in 2017-2018 in the city of Natal/RN, Brazil: a population-based survey

**DOI:** 10.1590/S2237-96222024v33e20231307.especial2.en

**Published:** 2025-01-10

**Authors:** Nayre Beatriz Martiniano de Medeiros, Isabelle Ribeiro Barbosa, Rita Barradas Barata, Ana Paula França, Ione Aquemi Guibu, José Cássio de Moraes, Carla Magda Allan Santos Domingues, Maria da Gloria Teixeira, Ana Clara Dantas de Souza, Eliene Roberta Alves Dos Santos, Mayonara Fabíola Silva Araújo, Clebson Verissimo da Costa Pereira, Ricardo Andrade Bezerra, Arthur Alexandrino, Fábia Cheyenne Gomes de Morais Fernandes, ICV 2020 Group, Adriana Ilha da Silva, Alberto Novaes Ramos, Ana Paula França, Andrea de Nazaré Marvão Oliveira, Antonio Fernando Boing, Carla Magda Allan Santos Domingues, Consuelo Silva de Oliveira, Ethel Leonor Noia Maciel, Ione Aquemi Guibu, Isabelle Ribeiro Barbosa Mirabal, Jaqueline Caracas Barbosa, Jaqueline Costa Lima, José Cássio de Moraes, Karin Regina Luhm, Karlla Antonieta Amorim Caetano, Luisa Helena de Oliveira Lima, Maria Bernadete de Cerqueira Antunes, Maria da Gloria Teixeira, Maria Denise de Castro Teixeira, Maria Fernanda de Sousa Oliveira Borges, Rejane Christine de Sousa Queiroz, Ricardo Queiroz Gurgel, Rita Barradas Barata, Roberta Nogueira Calandrini de Azevedo, Sandra Maria do Valle Leone de Oliveira, Sheila Araújo Teles, Silvana Granado Nogueira da Gama, Sotero Serrate Mengue, Taynãna César Simões, Valdir Nascimento, Wildo Navegantes de Araújo

**Affiliations:** 1 Universidade Federal do Rio Grande do Norte, Programa de Pós-Graduação em Saúde Coletiva, Santa Cruz, RN, Brazil; 2 Santa Casa de São Paulo, Faculdade de Ciências Médicas, São Paulo, SP, Brazil; 3 Organização Pan-Americana da Saúde, Brasília, DF, Brazil; 4 Universidade Federal da Bahia, Instituto de Saúde Coletiva, Salvador, BA, Brazil; 5 Universidade Federal do Rio Grande do Norte, Programa de Pós-Graduação em Saúde Coletiva, Natal, RN, Brazil; 6 Fundação Oswaldo Cruz, Bio-Manguinhos, Rio de Janeiro, RJ, Brazil; Universidade Federal do Espírito Santo, Vitória, ES, Brazil; Universidade Federal do Ceará, Departamento de Saúde Comunitária, Fortaleza, CE, Brazil; Faculdade Ciências Médicas Santa Casa de São Paulo, São Paulo, SP, Brazil; Secretaria de Estado da Saúde do Amapá, Macapá, AP, Brazil; Universidade Federal de Santa Catarina, SC, Brazil; Organização Pan-Americana da Saúde, Brasília, DF, Brazil; Instituto Evandro Chagas, Belém, PA, Brazil; Faculdade de Ciências Médicas Santa Casa de São Paulo, Departamento de Saúde Coletiva, São Paulo, SP, Brazil; Universidade Federal de Mato Grosso, Cuiabá, MT, Brazil; Universidade Federal do Paraná, Curitiba, PR, Brazil; Universidade Federal de Goiás, Goiânia, GO, Brazil; Universidade Federal do Piauí, Teresina, PI, Brazil; Universidade de Pernambuco, Faculdade de Ciências Médicas, Recife, PE, Brazil; Instituto de Saúde Coletiva, Universidade Federal da Bahia, Salvador, BA, Brazil; Secretaria de Estado da Saúde de Alagoas, Maceió, AL, Brazil; Universidade Federal do Acre, Rio Branco, AC, Brazil; Universidade Federal do Maranhão, Departamento de Saúde Pública, São Luís, MA, Brazil; Universidade Federal de Sergipe, Aracaju, SE, Brazil; Secretaria Municipal de Saúde, Boa Vista, RR, Brazil; Fundação Oswaldo Cruz, Mato Grosso do Sul, Campo Grande, MS, Brazil; Fundação Oswaldo Cruz, Escola Nacional de Saúde Pública Sergio Arouca, Rio de Janeiro, RJ, Brazil; Universidade Federal do Rio Grande do Sul, Porto Alegre, RS, Brazil; Fundação Oswaldo Cruz, Instituto de Pesquisa René Rachou, Belo Horizonte, MG, Brazil; Secretaria de Desenvolvimento Ambiental de Rondônia, Porto Velho, RO, Brazil; Universidade de Brasília, Brasília, DF, Brazil

**Keywords:** Programas de Inmunización, Cobertura de Vacunación, Encuestas Epidemiológicas, Inmunización, Immunization Programs, Vaccination Coverage, Health Surveys, Immunization

## Abstract

**Objective:**

To estimate vaccination coverage and analyze factors associated with full vaccination among children up to 15 months old in the city of Natal-RN, Brazil.

**Methods:**

Population-based survey with data recorded on children’s vaccination cards and interviews conducted in 2020 and 2021. Analysis of factors associated with complete vaccination was performed by calculating prevalence ratios (PR) and 95% confidence intervals (95%CI) using Poisson regression.

**Results:**

Among 688 children studied, vaccination coverage was 45.4% (95%CI 37.2;53.9) and 15.5% (95%CI 10.6;22.2) for valid and on-time doses, respectively. Higher vaccination coverage was associated with females (PR=1.08; 95%CI 0.78;1.48) and socioeconomic strata C and D (PR=1.44; 95%CI 1.03;2.02).

**Conclusions:**

The results demonstrate that the city of Natal has low vaccination coverage for all immunobiologicals.

## Study contributions

**Main results:**

The results showed vaccination coverage below the recommended target of 90% and a low percentage of on-time doses. The lowest socioeconomic strata (C and D) had higher vaccination coverage compared to the highest strata (A and B).

**Implications for services:**

The results can guide services in the development of strategies that inform the population about the importance of immunization, especially women who work and live with their partners and families from higher economic classes.

**Perspectives:**

Public policies need to be developed aimed at addressing low vaccination coverage indicators among children. Encouraging the development of studies to enable better monitoring of this data is also essential.

## RESUMEN

**Objetivo:**

Estimar la cobertura de vacunación y analizar los factores asociados a la vacunación completa hasta los 15 meses de vida en la ciudad de Natal, Rio Grande do Norte, Brasil.

**Métodos:**

Encuesta poblacional con datos registrados en la cartilla de vacunación y entrevistas realizadas en 2020 y 2021. El análisis de los factores asociados a la vacunación completa se realizó mediante el cálculo de razones de prevalencia (RP) e intervalos de confianza del 95% (IC 95%) por regresión de Poisson.

**Resultados:**

Entre los 688 niños estudiados, la cobertura de vacunación fue del 45,4% (IC95% 37,2;53,9) y del 15,5% (IC95% 10,6;22,2) para dosis válidas y oportunas, respectivamente. La mayor cobertura de vacunación se asoció con el sexo femenino (RP=1,08; IC 95% 0,78;1,48) y con los estratos socioeconómicos C y D (RP=1,44; IC 95% 1,03;2,02).

**Conclusione:**

Los resultados demuestran que la ciudad de Natal tiene baja cobertura de vacunación para todos los inmunobiológicos.

## INTRODUCTION

The Brazilian National Immunization Program (*Programa Nacional de Imunizações* - PNI) was created in 1973 with the aim of controlling the transmission of infectious diseases and reducing child mortality. It has established itself as one of the best programs in the world, recognized nationally and internationally for its effectiveness, the number of vaccines included on its schedule and its logistical and technological innovation capacity.^1^ The Program completed 50 years in 2023, and a large part of this success is due to the fact that the PNI follows the Brazilian National Health System (*Sistema* Único *de Saúde* – SUS) principles of universality, equity and decentralization.²

According to the PNI Information System, vaccination coverage of immunobiologicals recommended by the vaccination schedule for children up to 1 year old was 82.7% in 2017. Rio Grande do Norte is one of the Brazilian states that have vaccination coverage below the average national rate of 66.1%.^3^ An example of the drop in vaccination coverage in the Rio Grande do Norte is the poliovirus vaccine, the coverage of which fell from 90.0% of the target population in 2015, to 57.0% and 65.6% in 2017 and 2018, respectively.^3^

Research shows that the increase in the spread of fake news on the internet about supposed side effects of vaccines negatively impacts vaccination coverage.^4^ Socioeconomic factors, such as low maternal education levels, low family income and greater number of children, have also contributed to the decrease in child vaccination coverage.^4,5^ More recent studies demonstrate a change in the scenario of the drop in childhood vaccination coverage, being more pronounced in higher socioeconomic strata and among those with a high level of education, thus becoming a relevant issue for public health.^6,7^

Despite the PNI’s vaccination campaign efforts, the drop in vaccination coverage is a phenomenon observed throughout Brazil, highlighting the need for studies that evaluate and identify factors related to low coverage. As the logistics of vaccine administration are different, and there is a scarcity of studies that analyze child vaccination coverage in Natal, it is necessary gain an understanding of coverage profiles in order to enable effective formulation of effective public health policies that contribute to increasing vaccination coverage. The objective of this study was to estimate vaccination coverage in children up to 15 months old and analyze factors associated with full vaccination in Natal, Rio Grande do Norte, Brazil. 

## METHODS

### Study design 

This was a household-based survey based on the cohort of live births in the period 2017-2018, using cluster research methodology. It started with the children’s vaccination trajectory from birth until the moment of the interview, using records made on their vaccination cards. 

The data used came from the part of the National Vaccination Coverage Survey conducted in Natal, Rio Grande do Norte. The national survey covered the 26 Brazilian state capitals and Federal District. The survey was carried out by the Augusto Leopoldo Ayrosa Galvão Study Center of the *Santa Casa de São Paulo* Faculty of Medical Sciences Department of Social Medicine, in partnership with several universities, and with support from several state and municipal health departments.^8^

### Participants

The selected population was made up of children born alive in 2017 and 2018, living in the urban area of ​​Natal. According to data from the Demographic Census carried out in 2022 by the Brazilian Institute of Geography and Statistics (*Instituto Brasileiro de Geografia e Estatística*), Natal covers an area of 167,401m^2^, 99.3% of which is urbanized, and has a population of 751,300 inhabitants. The population’s average income is three minimum wages, however 35.7% of the population only has a per capita income of up to half a minimum wage.^9^ According to data from the Natal City Government, the municipality has 147 health services, of which 80 are municipal, 10 are state and 4 are federal public services. The municipality also counts on 47 outsourced private services and 6 outsourced charity services that assist the population via the SUS.^10^

The sample size was defined based on calculations adopted by the World Health Organization (WHO) for vaccination coverage surveys. The parameters used were: 95% confidence; 70% expected vaccination coverage; and a design effect of 1.4. Twice the number of children needed were randomly selected in order to compensate for possible losses.

Sampling was carried out in three stages. The first stage involved stratification of census tracts classified as being urban by the 2010 Demographic Census, based on three socioeconomic variables: average income of heads of family, percentage of heads of family with income above 20 minimum wages and percentage of literate heads of family. Using cluster analysis, these variables were used to define four socioeconomic strata within the areas of residence (compositional ecological variable), where A = high, B = medium high, C = medium low and D = low. 

In the second stage, census tract clusters were formed, containing one or more tracts. This was done according to the estimated number of live births from the 2017 cohort living in each of the census tracts, so that each cluster contained at least 56 live births. 

The data source for live births was the Live Birth Information System (*Sistema de Informação sobre Nascidos Vivos*), with nominal data on the child, mother and father and full address. These clusters were systematically selected at random in order for eight of them to be included in each stratum. This form of selection was used for two purposes: a methodological purpose, to increase the representativeness of the sample, including all regions of the city; and an operational purpose, to facilitate the carrying out of field work by not dispersing the attempts to locate the children throughout the entire city.

The third stage was to locate the children of the cohorts of interest, namely 2017 and 2018, living in the census tracts within the selected clusters, in order to include 226 children in each of the municipality’s strata, totaling 904 children.^8^

### Variables 

In this study, the dependent variable was full vaccination coverage of children up to 15 months old for the following vaccines: Bacillus Calmette-Guérin (BCG); hepatitis B; diphtheria, tetanus, pertussis, *Haemophilus influenzae* b and hepatitis B (DTP-Hib-HepB); pneumococcal conjugate (PCV10); poliovirus; rotavirus; meningococcal serogroup C (MENC); measles, mumps and rubella (MMR); hepatitis A; diphtheria, tetanus and pertussis (DTP); and varicella. 

Yellow fever vaccine was not considered in the calculations made by this study, because, according to the Ministry of Health, in Brazil vaccination against yellow fever is only mandatory in endemic places. Natal is not obliged to administer this immunobiological product. 

Full vaccination coverage was calculated taking the doses received in public and private services and recorded on the children’ vaccination cards. The numerator was the “number of children who completed the recommended vaccination schedule (BCG, hepatitis B, DTP-Hib-HepB [first, second and third dose, first booster], PCV10 [first and second dose, first booster], poliovirus [first, second and third dose, first booster], rotavirus [first and second dose], MENC [first and second dose, first booster], MMR [first and second dose], hepatitis A, DTP and varicella)”, divided by the denominator “number of children included in the study”, multiplied by 100.

Full vaccination coverage was classified into administered doses, valid doses and on-time doses. Valid doses were considered to be those administered between the minimum and maximum ages for dose administration, as well as the appropriate interval between doses for vaccines with a multi-dose schedule. On-time doses were those administered at the recommended age, that is, by the day before the child turned one month old, respecting the minimum age and intervals between doses. Coverage levels for the full basic scheme applied refer to all doses of vaccine received without making considerations about appropriate intervals between doses and the age of the child at the time of administration. 

The independent variables were as follows.

- Child’s characteristics: birth order (first, second, third, above third), child’s sex (female, male), race/skin color (White, Black, mixed race) and attending or having attended daycare and/or nursery (yes, no).
- Maternal characteristics: schooling in years (<8; 9-12; 13-15; ≥16), maternal age years (<20; 20-34; ≥35), maternal race/skin color (White, Black, mixed race), maternal job (yes, no) and marital status (has a partner, does not have a partner).
- Family characteristics: household crowding (yes – more than 3 household dwellers per room used as a bedroom, no – up to 3 household dwellers per room used as a bedroom), receives *Bolsa Família* benefit (yes, no), grandmother lives in household (yes, no), monthly family income (<BRL 1000; BRL 1001-3000; BRL 3001-8000; ≥BRL 8000; unable to answer or did not answer), socioeconomic stratum (strata A and B, strata C and D) and has used or uses private vaccination service (yes, no).

### Data analysis

As this study used complex sampling to obtain the data to be analyzed, the sample weight and the effect of the sample design were taken into consideration when calculating the prevalence of the outcome in relation to the independent variables. Distribution analysis took place by calculating the crude PRs, with respective 95% confidence intervals (95%CI), using Poisson regression. Statistical analysis was performed with Stata, version 13 (Stata Corp., College Station, United States).

### Ethical aspects

The survey was approved by the Research Ethics Committee of the *Instituto de Saúde Coletiva da Universidade Federal da Bahia*, as per Opinion No. 3.366.818, on June 4, 2019, and Certificate of Submission for Ethical Appraisal (*Certificado de Apresentação de Apreciação Ética* - CAAE) No. 4306919.5.0000.5030; and by the Research Ethics Committee of the *Irmandade da Santa Casa de São Paulo*, as per Opinion No. 4.380.019, on November 4, 2020, and CAAE No. 39412020.0.0000.5479. 

## RESULTS

The initial sample for Natal was 904 interviews, divided equally between four socioeconomic strata (224 interviews per stratum). The final sample consisted of 688 children (76.1%), resulting in a sample loss of 23.9%. Of the total number of children assessed, 53.1% were female, 61.0% were of mixed race/skin color, 56.1% had their grandmother living with them in the same household, 49.7% used private vaccination services, 52.0% were receiving or had received the *Bolsa Família* Program benefit, 49.1% of the mothers had a job, 75.7% of the mothers were under 20 years old and 61.3% were from socioeconomic strata C and D.

**Figure 1:**
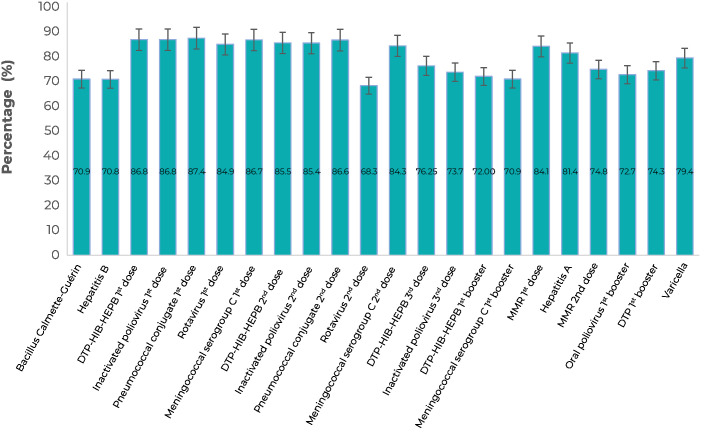
Vaccination coverage (administered doses) according to the vaccination schedule for children up to 15 months old, National Vaccination Coverage Survey, Natal, Rio Grande do Norte, Brazil, 2017-2018 (n=688)

Vaccination coverage in Natal was below 90.0% for all immunizing agents. Vaccination coverage is described in Figure 1 according to the vaccination schedule for children up to 15 months old. In this analysis of the immunobiologicals to be administered, we found that the BCG, hepatitis B and rotavirus vaccines, as well as the meningococcal C vaccine booster, had the lowest vaccination coverage when compared to the remainder.

**Figure 2:**
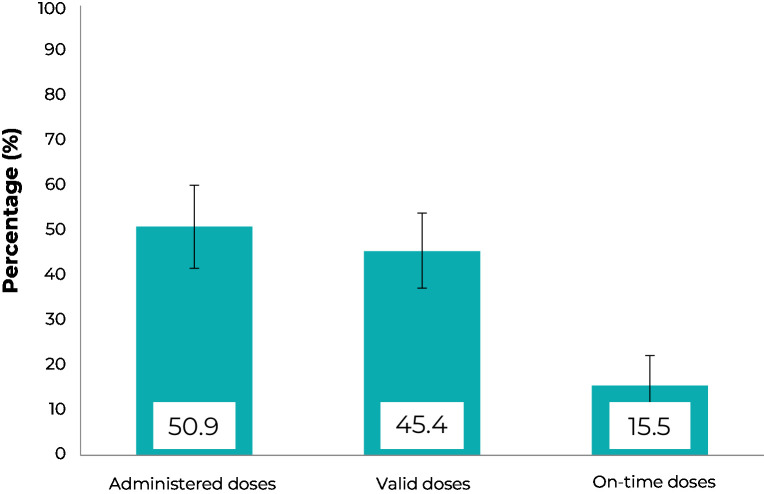
Full coverage of vaccines to be administered by 15 months old (except yellow fever vaccine), administered doses, valid doses and on-time doses, National Vaccination Coverage Survey, Natal, Rio Grande do Norte, Brazil, 2017-2018 (n=688)

Regarding comparison of full coverage, with regard to vaccines that should be administered up to 15 months old, we found 50.9% coverage (95%CI 41.6;60.1) for administered doses, 45. 4% (95%CI 37.2;53.9) for valid doses and 15.5% (95%CI 10.6;22.2) for on-time doses (Figure 2).

As for the analysis of full coverage of administered doses, for vaccines to be taken in the first 15 months of life, regarding the children’s characteristics (Table 1), maternal characteristics (Table 2) and family characteristics (Table 3), it is noteworthy that there is greater coverage among female children, accounting for 53.1% (PR=1.08; 95%CI 0.78;1.48), children of Black race/skin color, accounting for 51.1% (PR=1.11 ; 95%CI 0.56;2.17) and those of mixed race, accounting for with 61% (PR=1.32; 95%CI 0.87;2.01). 

**Table 1:** Full coverage of vaccines to be administered by 15 months old (except yellow fever vaccine), administered doses, valid doses and on-time doses, National Vaccination Coverage Survey, Natal, Rio Grande do Norte, Brazil, 2017-2018 (n=688)

Variables	Coverage % (95%CI)	**Crude PR (95%CI)**
**Birth order**		
First	48.5 (36.0;61.3)	1
Second	52.2 (35.7;68.2)	1.07 (0.70;1.64)
Third	64.0 (45.3;79.3)	1.31(0.90;1.93)
Above third	51.7 (34.7;68.3)	1.06 (0.71;1.57)
**Child’s sex**		
Male	49.0 (37.3;60.8)	1
Female	53.1 (40.6;65.3)	1.08 (0.78;1.48)
**Child’s race/skin color**		
White	45.9 (33.0;59.5)	1
Black	51.1 (23.4;77.8)	1.11 (0.56;2.17)
Mixed race	61.0 (47.2;73.2)	1.32 (0.87;2.01)
**Attends nursery/daycare**		
Yes	51.7 (39.4;63.5)	1
No	50.2 (34.2;66.2)	0.97 (0.62;1.50)

**Table 2:** Descriptive and bivariate analysis showing prevalence ratios (PR) and 95% confidence intervals (95%CI) of the full vaccination coverage of vaccines recommended for children up to 15 months old (except yellow fever), based on administered doses, according to maternal characteristics, Vaccination Coverage Survey, Natal, Rio Grande do Norte, Brazil, 2017-2018 (n=688)

Variables	Coverage % (95%CI)	Crude PR (95%CI)
**Schooling (years)**		
≤8	50.8 (38.2;63.3)	1
9-12	76.7 (59.7;87.9)	1.51 (1.16;1.96)
13-15	55.3 (42.2;67.7)	1.08 (0.75;1.56)
≥16	45.8 (30.9;61.5)	0.90 (0.59;1.37)
**Maternal age (years)**		
<20	75.7 (31.9;95.3)	1
20-34	48.6 (37.7;59.6)	0.64 (0.38;1.06)
≥35	53.1 (40.2;65.5)	0.70 (0.41;1.17)
**Maternal race/skin color**		
White	43.9 (27.1;62.2)	1
Black	62.6 (47.6;75.5)	1.42 (0.88;2.29)
Mixed race	58.9 (45.6;71.1)	1.34 (0.76;2.34)
**Maternal job**		
Yes	49.1 (35.9;62.5)	1.18 (0.87;1.60)
No	58.2 (50.2;65.7)	1
**Marital status**		
Has a partner	55.6 (45.1;65.7)	0.84 (0.47;1.50)
Does not have a partner	46.8 (26.0;68.8)	1

**Table 3:** Descriptive and bivariate analysis showing prevalence ratio (PR) and 95% confidence intervals (95%CI) of full vaccination coverage of vaccines to be administered up to 15 months old (except yellow fever), based on administered doses, according to family characteristics, Vaccination Coverage Survey, Natal, Rio Grande do Norte, Brazil, 2017-2018 (n=688)

Variables	Coverage % (95%CI)	Crude PR (95%CI)
**Household crowding**		
Yes (above 4 people/bedroom)	51.8 (41.6;61.9)	0.70 (0.36;1.35)
No (0-3 people/bedroom)	36.6 (18.4;59.6)	1
**Receives** *Bolsa* *Família* **benefit**		
Yes	52.0 (38.6;65.2)	1
No	50.5 (38.9;62.0)	0.97 (0.68;1.37)
**Grandmother lives in household**		
Yes	56.1 (38.7;72.1)	1
No	48.4 (35.4;61.6)	0.86 (0.54;1.37)
**Monthly family income (BRL)**		
<1000	57.8 (50.7;64.6)	1
1001-3000	62.2 (45.7;76.4)	1.07 (0.81;1.41)
3001-8000	64.2 (34.7;85.8)	1.11 (0.70;1.74)
≥8000	40.5 (8.5;83.4)	0.70 (0.21;2.31)
Unable to answer/did not answer	46.8 (23.1;72.2)	0.59 (0.33;1.06)
**Socioeconomic stratum**		
Strata A and B	42.4 (29.6;56.2)	1
Strata C and D	61.3 (54.7;67.4)	1.44 (1.03;2.02)
**Use of private vaccination service**		
No	54.0 (29.2;77.0)	1
Yes	49.7 (37.4;62.0)	0.91 (0.49;1.69)

Vaccination coverage was higher for the following variables: children born to women with between 9 and 12 years of schooling, accounting for 76.7% (PR=1.51; 95%CI 1.16;1.96); mothers under 20 years old, 75.7% (PR=1; 95%CI); mothers of Black race/skin color, 62.6% (PR=1.42; 95%CI 0.88;2.29), mothers who did not have a job, 58.2% (PR=1; 95%CI); mothers who had a partner, 55.6% (PR=0.84; 95%CI 0.47;1.50); families were receiving or had received *Bolsa Família* Program benefit, 52% (PR =1; 95%CI); children with a grandmother living in the same household, 56.1% (PR=1; 95%CI); children in socioeconomic strata C and D, 61.3% (PR=1.44; 95%CI 1.03;2.02); not using private vaccination services, 54% (PR=1; 95%CI).

In the bivariate analysis, vaccination coverage was higher for the following variables: mothers with between 9 and 12 years of schooling (PR=1.51; 95%CI 1.16;1.96) (Table 2), in particular for children from socioeconomic strata C and D (PR=1.44; 95%CI 1.03;2.02) (Table 3).

## DISCUSSION

The results of this research showed that full vaccination coverage for children up to 15 months old in Natal was below the recommended PNI targets. The data is of greater concern with regard to the low percentages of on-time doses. 

A study carried in Rondonópolis, in the Brazilian state of Mato Grosso, assessed coverage of vaccines recommended for the vaccination schedule for children up to 12 months old. That study identified on-time coverage below the 90.0% PNI target, corroborating the results of our study.^11^

The alert about the process of falling vaccination coverage in Brazil was also described by Barbieri, who in 2017 identified that only the BCG vaccine achieved the recommended PNI target. In 2019, coverage for all immunobiologicals on the Brazilian childhood vaccination schedule was below the recommended target.^12^ This phenomenon is also occurring in other countries. The Estimates of National Immunization Coverage report, prepared in 2019 by the WHO and the United Nations Children’s Fund, highlights that among the 193 WHO Member States coverage did not achieve 90% for any of the vaccines recommended on the child vaccination schedule.^13,14,15^

Vaccines enable eradication and control of infectious diseases, such as polio and smallpox, as well as reduction of child mortality. Recently, vaccines prevented the deaths of more than 20 million people during the COVID-19 pandemic. Despite the proven and unquestionable effectiveness of vaccines, there is a global trend towards reducing vaccination coverage, which creates risks for the reemergence of diseases that had been eradicated and controlled.^16,17^

An ecological study carried out in the Brazilian state of Paraíba highlighted the low vaccination coverage of some of these immunobiologicals, especially BCG.^18^ Inconsistency in the availability of immunobiologicals in primary care services may also have influenced the low coverage. It is important to highlight that the Ministry of Health published an informative note in 2017 about the shortage of BCG and hepatitis B vaccines. This could have a direct impact on on-time vaccination, since these immunobiologicals are administered at birth.^19^

Irregular supply and availability of vaccines in primary care services are factors that directly impact vaccination coverage. A study published in 2018 analyzed the trend in vaccine availability in Brazil in 2012, 2014 and 2018. It identified that, despite there being a growing trend in the availability of all vaccines investigated, around a quarter of Brazilian primary health care centers did not have all vaccines available in 2018. Shortages, even in the short term, weaken continuity of care and can cause harm regarding trust in the PNI.^20^

A study conducted by Baralhas et al.,^21^ in a city in the Brazilian state of São Paulo, identified that families with better financial conditions refuse to accept home visits by community health agents and believe that they do not need to register with the SUS, due to the fact that they have private health insurance. The analyses performed by our study demonstrate and highlight this direct socioeconomic impact on accessing public health services. 

Some studies have associated low parental education, lower income and Black maternal race/skin color as determining factors for low immunization.^22,23^ The results of our study confirm a change in the global scenario, already described in more recent studies that demonstrate a greater risk of incomplete vaccination as per the schedule among families from higher economic classes.^6,7^ Identifying changes in these associated factors is of great importance for informing the formulation of public health policies. 

This study identified that there is lower vaccination coverage among children whose mothers work, although there was no statistical significance. This result may be related to vaccination room opening hours. A study^25^ identified that restricting vaccination room opening hours is a barrier to health service access, given that a large part of the population works and has less time to go in search of vaccination services that are open when they need them. The authors emphasize that some vaccination rooms do not stay open for as long as primary health care centers do. There are vaccination rooms that only open in the afternoon, and others only once a week, for example. 

In 2019, the Ministry of Health announced a plan containing measures to address low vaccination coverage. The ten steps identified to ensure the expansion of vaccination coverage include measures such as keeping vaccination rooms open throughout health center opening hours, not being required to present proof of address at the time of vaccination, using medical appointments and appointments with other health professionals to advise on keeping vaccination up to date, monitoring health service users whose vaccinations are overdue, as well actively tracing them.^27^

The drop in vaccination coverage goes hand-in-hand with an in-depth discussion about mandatory vaccination of children, provided for by the Child and Adolescent Statute, Law No. 8069/1990, article 14, paragraph 1, “vaccination of children is mandatory in cases recommended by health authorities”.^28^ Many parents and guardians choose not to vaccinate their children, so some municipalities, in search of solutions for low vaccination coverage, have enacted laws that require presentation of an updated vaccination record as a condition for enrollment of children at school. The Brazilian state of Ceará enacted Law No. 16929/2019 which requires presentation of an updated vaccination card for enrollment at public and private primary or secondary schools.^29^ In Natal, despite a public notice having been published requesting proof of vaccination at the time of school enrollment, this is not mandatory. 

Standing out among the limitations of this study are study participant information and memory bias, which may also have influenced the results. There was a high percentage of refusal to participate in the study among families from economic strata A (60.4%) and B (47.5%). It is noteworthy that analysis of vaccination coverage, with reliable data, is an important instrument for assessing the characteristics of a disease in the population and for establishing more effective actions to control potentially preventable diseases.

In the context of public health, in particular, the results found by this study can enable health managers at municipal, state and federal levels to act together with regard to logistics in order to avoid delays and shortages of immunobiologicals in vaccination rooms, as well as to plan actions capable of increasing the public’s adherence to childhood vaccination. The results of this study highlight the importance of health education for parents and other people responsible for children, in order to promote knowledge and recognition of vaccination being essential. Measures such as intensifying the dissemination of the vaccination schedule through digital media, facilitating access to health services, with more flexible vaccination room opening times, formulating and implementing health policies that promote the strengthening of health surveillance and frequently carrying out household surveys can contribute to increasing vaccination coverage in children up to 15 months old. 

It is important to emphasize that health actions must be intended to ensure universal access for the entire population. The aim is to guarantee the achievement of targets that have been set, as these are important for preventing and eradicating preventable diseases, and consequently for avoiding the reemergence of eradicated diseases that ravaged the world in times gone by. 

## References

[1] Domingues CMAS, Maranhão AGK, Teixeira AM, Fantinado FFS, Domingues RAS (2020). 46 anos do Programa Nacional de Imunizações: uma história repleta de conquistas e desafios a serem superados. Cad Saúde Pública.

[2] Pinheiro LV, Júnior FPD, Silva IM, Câmara JGA, Torres LA, Maia ARF N (2021). O alvorecer da imunização no século XXI. Científic@ Multidisciplinary Journal.

[3] Ministério da Saúde (BR) (2023). Banco de dados do Sistema Único de Saúde-DATASUS.

[4] Césare N, Mota TF, Lopes FFL, Lima ACM, Luzardo R, Quintanilha LF (2020). Longitudinal profiling of the vaccination coverage in Brazil reveals a recent change in the patterns hallmarked by differential reduction across regions. Int J Infect Dis.

[5] Yokokura AVCP, Silva AAM, Bernardes ACF, Filho FL, Alves MTSSB, Cabra NAL MF (2013). Cobertura Vacinal e fatores associados ao esquema vacinal básico incompleto aos 12 meses de idade, São Luís, Maranhão, Brasil, 2006. Cad Saúde Púb.

[6] Silveira MF, Buffarini R, Bertoldi AD, Santos IS, Barros AJD, Matijasevich A R (2020). The emergence of vaccine hesitancy among upper-class Brazilians: results from four birth cohorts, 1982–2015. Vac.

[7] Buffarini R, Barros FC, Silveira MF (2020). Vaccine coverage within the first year of life and associated factors with incomplete immunization in a Brazilian birth cohort. Arch Pub Health.

[8] Barata RB, França AP, Guibu IA, Vasconcellos MTL, Moraes JC, Grupo ICV Natal (2023). Inquérito Nacional de Cobertura Vacinal 2020: métodos e aspectos operacionais. Rev Bras Epidemiol.

[9] Instituto Brasileiro de Geografia e Estatística (IBGE) (2024). Instituto Brasileiro de Geografia e Estatística (IBGE).

[10] Prefeitura Municipal do Natal (2007). (Re)desenhando a rede de saúde na cidade do Natal/Secretaria Municipal de Saúde de Natal.

[11] Lemos PL, Júnior GJO, Souza NFC, Silva IM, Paula IPG, Silva KC CLA (2022). Fatores associados ao esquema vacinal incompleto. Rev Paul Pediatr.

[12] Barbieri CLA (2021). Imunização e cobertura vacinal: passado, presente e futuro.

[13] World (2019). Estimates of National Immunization Coverage. Are we losing ground?.

[14] World Health Organization (2020). Immunization coverage.

[15] World Health Organization (2020). Global polio eradication initiative applauds WHO african region for wild polio-free certifcation.

[16] Souza MCC (2023). Adesão à imunização infantil no Brasil: uma revisão narrativa. Sci Elec Arch.

[17] Pecetta S, Nandi A, Weller C, Harris V, Fletcher H, Scorza FB NSP (2023). Vaccines for a sustainable planet. Sci Transl Med.

[18] Cunha NSP, Fahrat SCL, Olinda RA, Braga ALF, Barbieri CLA, Pamplona YAP, Martins LC (2022). Spatial analysis of vaccine coverage on the first year of life in the northeast of Brazil. BMC Public Health.

[19] Ministério da Saúde (BR) (2017). Nota Informativa Nº 134-SEI/2017-CGPNI/DEVIT/SVS/MS.

[20] Neves RG (2022). Tendência da disponibilidade de vacinas no Brasil: PMAQ-AB 2012, 2014 e 2018. Cad Saúde Púb.

[21] Baralhas M, Pereira MAO (2013). Prática diária dos agentes comunitários de saúde: dificuldades e limitações da assistência. Rev Bras Enferm.

[22] Yokokura AVCP, Silva AAM, Bernardes ACF, Filho FL, Alves MTSSB, Cabra NAL (2013). Cobertura vacinal e fatores associados ao esquema vacinal básico incompleto aos 12 meses de idade, São Luís, Maranhão, Brasil, 2006. Cad Saúde Púb.

[23] Barata RB, Pereira SM (2013). Desigualdades sociais e cobertura vacinal na cidade de Salvador, Bahia. Rev Bras Epidemiol.

[24] Moraes JC, Ribeiro MCSA (2008). Social inequalities and vaccination coverage: utilization of household surveys. Rev Bras Epidemiol.

[25] Duarte DC, Viegas SMF, Augusto TFS, Oliveira VC, Martins JRT, Tholl AD (2021). Aspectos organizacionais e uma agenda para o acesso à vacinação sob a ótica do usuário. Texto & contexto enferm.

[26] Andrade VMP, Cardoso CL (2017). Visitas domiciliares de agentes comunitários de saúde: concepções de profissionais e usuários. Psico UFC.

[27] CONASEMS (2019). Dez passos para ampliar cobertura vacinal.

[28] Estatuto da Criança e do Adolescente (BR) (1990). Estatuto da Criança e do Adolescente (BR).

[29] Assembleia Legislativa do Ceará (2019). Dispõe sobre a obrigatoriedade da apresentação da carteira de vacinação no ato da matrícula e rematrícula escolar.

