# Supplementary material for: Factors associated with vaccination coverage in children up to 15 months old, born in 2017-2018 in the city of Natal/RN, Brazil: a population-based survey
Source: Epidemiol Serv Saude. 2025 Jan 10;33(spe2):e20231307. doi: 10.1590/S2237-96222024v33e20231307.especial2.en (PMC11734606; doi:10.1590/S2237-96222024v33e20231307.especial2.en)
Supplement: Translation

# Translation: Fatores associados às coberturas vacinais em crianças com até 15 meses de vida, nascidas no período 2017-2018 em Natal, Rio Grande do Norte: inquérito de base populacional

**DOI:** 10.1590/S2237-96222024v33e20231307.especial2.pt

**Authors:** Nayre Beatriz Martiniano de Medeiros, Isabelle Ribeiro Barbosa, Rita Barradas Barata, Ana Paula França, Ione Aquemi Guibu, José Cássio de Moraes, Carla Magda Allan Santos Domingues, Maria da Gloria Teixeira, Ana Clara Dantas de Souza, Eliene Roberta Alves Dos Santos, Mayonara Fabíola Silva Araújo, Clebson Verissimo da Costa Pereira, Ricardo Andrade Bezerra, Arthur Alexandrino, Fábia Cheyenne Gomes de Morais Fernandes, Grupo ICV 2020, Adriana Ilha da Silva, Alberto Novaes Ramos, Ana Paula França, Andrea de Nazaré Marvão Oliveira, Antonio Fernando Boing, Carla Magda Allan Santos Domingues, Consuelo Silva de Oliveira, Ethel Leonor Noia Maciel, Ione Aquemi Guibu, Isabelle Ribeiro Barbosa Mirabal, Jaqueline Caracas Barbosa, Jaqueline Costa Lima, José Cássio de Moraes, Karin Regina Luhm, Karlla Antonieta Amorim Caetano, Luisa Helena de Oliveira Lima, Maria Bernadete de Cerqueira Antunes, Maria da Gloria Teixeira, Maria Denise de Castro Teixeira, Maria Fernanda de Sousa Oliveira Borges, Rejane Christine de Sousa Queiroz, Ricardo Queiroz Gurgel, Rita Barradas Barata, Roberta Nogueira Calandrini de Azevedo, Sandra Maria do Valle Leone de Oliveira, Sheila Araújo Teles, Silvana Granado Nogueira da Gama, Sotero Serrate Mengue, Taynãna César Simões, Valdir Nascimento, Wildo Navegantes de Araújo

**Affiliations:** 1 Universidade Federal do Rio Grande do Norte, Programa de Pós-Graduação em Saúde Coletiva, Santa Cruz, RN, Brasil; 2 Santa Casa de São Paulo, Faculdade de Ciências Médicas, São Paulo, SP, Brasil; 3 Organização Pan-Americana da Saúde, Brasília, DF, Brasil; 4 Universidade Federal da Bahia, Instituto de Saúde Coletiva, Salvador, BA, Brasil; 5 Universidade Federal do Rio Grande do Norte, Programa de Pós-Graduação em Saúde Coletiva, Natal, RN, Brasil; 6 Fundação Oswaldo Cruz, Bio-Manguinhos, Rio de Janeiro, RJ, Brasil; Universidade Federal do Espírito Santo, Vitória, ES, Brasil; Universidade Federal do Ceará, Departamento de Saúde Comunitária, Fortaleza, CE, Brasil; Faculdade Ciências Médicas Santa Casa de São Paulo, São Paulo, SP, Brasil; Secretaria de Estado da Saúde do Amapá, Macapá, AP, Brasil; Universidade Federal de Santa Catarina, SC, Brasil; Organização Pan-Americana da Saúde, Brasília, DF, Brasil; Instituto Evandro Chagas, Belém, PA, Brasil; Faculdade de Ciências Médicas Santa Casa de São Paulo, Departamento de Saúde Coletiva, São Paulo, SP, Brasil; Universidade Federal do Rio Grande do Norte, Natal, RN, Brazil; Universidade Federal do Ceará, Departamento de Saúde Comunitária, Fortaleza, CE, Brazil; Universidade Federal de Mato Grosso, Cuiabá, MT, Brasil; Universidade Federal do Paraná, Curitiba, PR, Brasil; Universidade Federal de Goiás, Goiânia, GO, Brasil; Universidade Federal do Piauí, Teresina, PI, Brasil; Universidade de Pernambuco, Faculdade de Ciências Médicas, Recife, PE, Brasil; Instituto de Saúde Coletiva, Universidade Federal da Bahia, Salvador, BA, Brasil; Secretaria de Estado da Saúde de Alagoas, Maceió, AL, Brasil; Universidade Federal do Acre, Rio Branco, AC, Brasil; Universidade Federal do Maranhão, Departamento de Saúde Pública, São Luís, MA, Brasil; Universidade Federal de Sergipe, Aracaju, SE, Brasil; Secretaria Municipal de Saúde, Boa Vista, RR, Brasil; Fundação Oswaldo Cruz, Mato Grosso do Sul, Campo Grande, MS, Brasil; Fundação Oswaldo Cruz, Escola Nacional de Saúde Pública Sergio Arouca, Rio de Janeiro, RJ, Brasil; Universidade Federal do Rio Grande do Sul, Porto Alegre, RS, Brasil; Fundação Oswaldo Cruz, Instituto de Pesquisa René Rachou, Belo Horizonte, MG, Brasil; Secretaria de Desenvolvimento Ambiental de Rondônia, Porto Velho, RO, Brasil; Universidade de Brasília, Brasília, DF, Brasil

**Keywords:** Programas de Imunização, Cobertura Vacinal, Inquéritos Epidemiológicos, Imunização

## Abstract

**Objetivo:**

Estimar as coberturas vacinais e analisar os fatores associados à vacinação completa de crianças com até 15 meses de vida em Natal, Rio Grande do Norte.

**Métodos:**

Utilizou-se inquérito de base populacional com dados registrados na caderneta de vacinação e entrevistas realizadas em 2020 e 2021. A análise de fatores associados à vacinação completa foi realizada pelo cálculo das razões de prevalência (RP) e intervalos de confiança de 95% (IC_95%_) pela regressão de Poisson.

**Resultados:**

Entre as 688 crianças estudadas, a cobertura vacinal foi de 45,4% (IC_95%_ 37,2;53,9), e de 15,5% (IC_95%_ 10,6;22,2) para as doses válidas e oportunas. O sexo feminino (RP=1,08; IC_95%_ 0,78;1,48) e os estratos socioeconômicos C e D (RP=1,44; IC_95%_ 1,03;2,02) foram os que tiveram a maior cobertura vacinal.

**Conclusões:**

Os resultados mostraram baixas coberturas vacinais para todos os imunobiológicos em Natal.

## Contribuições do estudo

**Principais resultados:**

Os resultados mostraram coberturas vacinais abaixo da meta preconizada de 90% e baixo percentual de doses oportunas. Os estratos socioeconômicos mais baixos (C e D) apresentaram maiores coberturas vacinais comparadas com as dos estratos mais altos (A e B).

**Implicações para os serviços:**

Os resultados podem nortear os serviços para o desenvolvimento de estratégias que esclareçam a população sobre a importância da imunização, sobretudo em mulheres que trabalham e vivem com o companheiro e em famílias de classes econômicas mais altas.

**Perspectivas:**

Faz-se necessária a elaboração de políticas públicas direcionadas ao enfrentamento dos baixos indicadores de cobertura vacinal em crianças. O estímulo à elaboração de estudos para melhor monitoramento desses dados também é fundamental.

## INTRODUÇÃO

O Programa Nacional de Imunizações (PNI) foi criado em 1973 com o objetivo de controlar a transmissão de doenças infecciosas e reduzir a mortalidade infantil. Consolidou-se como um dos melhores programas do mundo, reconhecido nacional e internacionalmente por sua efetividade, pelo número de vacinas incluídas no seu calendário e pela capacidade logística e de inovação tecnológica.^1^ O programa completou 50 anos em 2023, e grande parte desse sucesso se deve ao fato de o PNI seguir os princípios da universalidade, equidade e descentralização do Sistema Único de Saúde (SUS).²

Segundo o Sistema de Informações do PNI, a cobertura vacinal dos imunobiológicos recomendados no calendário vacinal para crianças com até 1 ano de idade foi de 82,7% em 2017. O Rio Grande do Norte está entre os estados que apresentam cobertura vacinal abaixo da média brasileira de 66,1%.^3^ Um exemplo da queda na cobertura vacinal no estado pode ser observado para a vacina contra a poliomielite, cuja cobertura, de 90,0% da população-alvo em 2015, atingiu os percentuais de 57,0% e 65,6% em 2017 e 2018.^3^

Pesquisas demonstram que o aumento da propagação de notícias falsas a respeito de supostos efeitos colaterais das vacinas, por meio da internet, impactam a cobertura vacinal de forma negativa.^4^ Fatores socioeconômicos, como baixa escolaridade materna, baixa renda familiar e maior número de filhos, têm igualmente contribuído para a diminuição na cobertura vacinal de crianças.^4,5^ Estudos mais recentes demonstram uma mudança no cenário de queda na cobertura vacinal infantil, mais acentuada nos estratos socioeconômicos mais altos e de nível de escolaridade elevado, tornando-se uma questão relevante para a saúde coletiva, ^6,7^

Apesar dos esforços do PNI com campanhas de vacinação, a queda da cobertura vacinal é um fenômeno observado em todo o Brasil, ressaltando a necessidade de estudos que avaliem e identifiquem os fatores relacionados à baixa cobertura. Como a logística de aplicação da vacina é distinta, e há uma escassez de estudos que analisam a cobertura vacinal de crianças em Natal, é preciso compreender quais os perfis de cobertura para formulação efetiva de políticas públicas de saúde efetivas que colaborem para aumento na cobertura vacinal. O objetivo deste estudo foi estimar as coberturas vacinais até 15 meses de vida e analisar os fatores associados à vacinação completa em Natal-Rio Grande do Norte. 

## MÉTODOS

### Desenho do estudo

Tratou-se de um inquérito de base domiciliar a partir da coorte de nascidos vivos no período 2017-2018, baseado na metodologia de pesquisa por conglomerado. Partiu-se da trajetória de vacinação da criança desde o seu nascimento até o momento da entrevista, por meio de registros existentes nas cadernetas de vacinação. 

Os dados utilizados foram provenientes do Inquérito Nacional de Cobertura Vacinal em Natal-Rio Grande do Norte, através de uma pesquisa que abrangeu as 26 capitais brasileiras e o Distrito Federal. O estudo foi realizado pelo Centro de Estudos Augusto Leopoldo Ayrosa Galvão do Departamento de Medicina Social da Faculdade de Ciências Médicas da Santa Casa de São Paulo, em parceria com diversas universidades, com apoio de diversas secretarias estaduais e municipais de saúde.^8^

### PARTICIPANTES

A população selecionada foi composta por nascidos vivos em 2017 e 2018, residentes na área urbana de Natal. Segundo dados do Censo Demográfico realizado em 2022 pelo Instituto Brasileiro de Geografia e Estatística, Natal possui um território de 167,401 m^2^, com população de 751.300 habitantes e 99,3% de área urbanizada. O rendimento médio da população é de 3 salários mínimos, porém 35,7% da população possui renda *per capita* de até 0,5 salário mínimo.^9^ De acordo com dados da prefeitura de Natal, o município conta com 147 unidades de saúde, sendo 80 públicas municipais, 10 estaduais e 4 federais. Conta também com 47 unidades privadas contratadas e 6 filantrópicas que prestam serviço à população por meio do SUS.^10^

O tamanho da amostra foi definido a partir dos cálculos adotados pela Organização Mundial da Saúde (OMS) para inquéritos de cobertura vacinal. Utilizaram-se os parâmetros de 95% de confiança, uma cobertura vacinal esperada de 70% e efeito de desenho de 1,4. Foi sorteado o dobro de crianças para dar conta das possíveis perdas.

A amostragem foi realizada em três etapas. A primeira foi a estratificação dos setores censitários classificados como urbanos no Censo Demográfico 2010, com base em três variáveis socioeconômicas: renda média dos responsáveis pelas famílias, porcentagem de responsáveis com renda superior a 20 salários mínimos e porcentagem de responsáveis alfabetizados. Essas variáveis foram utilizadas para, através de uma análise de *cluster*, definir quatro estratos socioeconômicos das áreas de residência (variável ecológica composicional), sendo A igual a alto, B igual a médio alto, C igual a médio baixo e D igual a baixo. 

Na segunda etapa, formaram-se conglomerados de setores, contendo um ou mais setores. Isso foi feito de acordo com o número estimado de nascidos vivos da coorte de 2017 residentes em cada um dos setores censitários, de modo que cada conglomerado tivesse pelo menos 56 nascidos vivos. 

A fonte de dados dos nascidos vivos originou-se do Sistema de Informação sobre Nascidos Vivos, com dados nominais da criança, da mãe e do pai e endereço completo. Procedeu-se ao sorteio sistemático desses conglomerados de modo a incluir 8 destes em cada estrato. Esse sorteio foi feito com duas finalidades: uma metodológica, para aumentar a representatividade da amostra incluindo todas as regiões da cidade, e outra operacional, para facilitar a realização do trabalho em campo não dispersando a busca das crianças por todo o município.

A terceira etapa foi a localização das crianças das coortes de interesse, entre 2017 e 2018, residentes dos setores censitários pertencentes aos conglomerados sorteados, de modo a incluir 226 crianças em cada estrato do município, totalizando 904 crianças.^8^

### Variáveis 

Neste estudo foi considerada como variável dependente a cobertura vacinal completa de crianças com até 15 meses de vida para as vacinas: BCG, contra hepatite B, pentavalente, pneumocócica 10-valente (VPC10), contra poliomielite, contra rotavírus humano, meningocócica C (MenC), tríplice viral, contra hepatite A, contra difteria, tétano e pertússis (DTP) e contra varicela. 

A vacina contra febre amarela não foi considerada para cálculos deste estudo, porque, conforme o Ministério da Saúde, no Brasil a vacina contra febre amarela só é obrigatória em locais endêmicos. Natal não tem obrigatoriedade de aplicação desse imunobiológico. 

Para o cálculo da cobertura vacinal completa, consideraram-se as doses recebidas em serviços públicos e privados, constantes na caderneta de vacinação. Foi utilizado como numerador o “número de crianças que completaram o esquema vacinal preconizado (BCG, vacina contra hepatite B, vacina pentavalente [primeira, segunda e terceira dose, primeiro reforço], VPC10 [primeira e segunda dose, primeiro reforço], vacina contra poliomielite [primeira, segunda e terceira dose, primeiro reforço], vacina contra rotavírus humano [primeira e segunda dose], MenC [primeira e segunda dose, primeiro reforço], tríplice viral [primeira e segunda dose], vacina contra hepatite A, DTP e vacina contra varicela)”, dividido pelo denominador “número de crianças incluídas no estudo”, multiplicado por 100.

A cobertura vacinal completa foi classificada em doses aplicadas, doses válidas e doses oportunas. Consideraram-se como doses válidas aquelas aplicadas entre as idades mínimas e máximas de aplicação da dose, assim como o intervalo adequado entre as doses para vacinas com esquema multidose. As doses oportunas foram aquelas aplicadas na idade preconizada, ou seja, até o dia anterior ao que a criança completaria mais 1 mês de idade, respeitando-se a idade mínima e os intervalos entre as doses. As coberturas referentes ao esquema básico completo aplicado referem-se a todas as doses de vacina recebidas sem considerações referentes aos intervalos apropriados entre as doses e a idade da criança no momento da aplicação. 

As variáveis independentes foram as elencadas a seguir.

- Características da criança: ordem de nascimento (primeiro, segundo, terceiro, acima do terceiro), sexo da criança (feminino, masculino), raça/cor da pele (branco, preto, pardo) e frequenta ou frequentou a creche e berçário (sim, não).
- Características da mãe: anos de estudo em anos (<8; 9-12; 13-15; ≥16), idade da mãe em anos (<20; 20-34; ≥35), raça/cor da pele da mãe (branca, preta, parda), trabalho materno (sim, não) e situação conjugal (possui companheiro, não possui companheiro).
- Características familiares: aglomeração intradomiciliar (sim – mais de 3 pessoas residentes por cômodo utilizado para dormitório, não – até 3 pessoas residentes por cômodo utilizado para dormitório), recebe Bolsa Família (sim, não), presença da avó no domicílio (sim, não), renda familiar mensal (<R$ 1.000,00; R$ 1.001,00-R$ 3.000,00; R$ 3.001,00-R$ 8.000,00; ≥R$ 8.000,00; não sabe ou não respondeu), estrato socioeconômico (estrato A e B, estrato C e D) e fez ou faz uso de serviço privado para vacinação (sim, não).

### Análise de dados

Por se tratar de um estudo com amostragem complexa para as análises de dados, consideraram-se o peso da amostra e o efeito do desenho da amostra no cálculo da prevalência do desfecho em relação às variáveis independentes. A análise das distribuições procedeu com o cálculo da RP bruta, com respectivos intervalos de confiança de 95% (IC_95%_), por meio de regressão de Poisson. A análise estatística utilizou o programa Stata, versão 13 (Stata Corp., College Station, United States).

### Aspectos éticos

O inquérito foi aprovado pelos comitês de ética em pesquisa com seres humanos do Instituto de Saúde Coletiva da Universidade Federal da Bahia, sob Parecer nº 3.366.818, em 4 de junho de 2019, com Certificado de Apresentação de Apreciação Ética (CAAE) 4306919.5.0000.5030; e da Irmandade da Santa Casa de São Paulo, sob Parecer nº 4.380.019, em 4 de novembro de 2020, com CAAE 39412020.0.0000.5479. 

## RESULTADOS

A amostra inicial para Natal foi de 904 entrevistas, divididas igualmente entre quatro estratos socioeconômicos (224 entrevistas por estrato). A amostra final foi composta por 688 crianças (76,1%), resultando em uma perda amostral de 23,9%. Do total de crianças avaliadas, 53,1% são do sexo feminino, 61,0% têm cor da pele parda, 56,1% têm uma das avós morando no domicílio, 49,7% utilizaram serviços privados de vacinação, 52,0% recebem ou já receberam Bolsa Família, 49,1% das mães trabalham, 75,7% das mães têm faixa etária abaixo de 20 anos e 61,3% são das classes de consumo C e D.

Natal apresentou estimativa de cobertura vacinal abaixo de 90,0% para todos os imunizantes. Na Figura 1, estão descritas as coberturas vacinais segundo vacinas do calendário vacinal para crianças com até 15 meses de vida**.** Na análise isolada por imunobiológico, observa-se que as vacinas BCG, contra hepatite B e contra rotavírus humano e o reforço da vacina meningocócica C apresentaram as mais baixas coberturas vacinais quando comparadas com as demais.

**Figura 1:** Coberturas vacinais (doses aplicadas) segundo vacinas do calendário vacinal para crianças com até 15 meses de vida, Inquérito Nacional de Cobertura Vacinal, Natal, Rio Grande do Norte, 2017-2018 (n=688)

Quanto à comparação das coberturas completas, referente às vacinas que deveriam ser aplicadas até os 15 meses, nota-se o percentual de cobertura de 50,9% (IC_95%_ 41,6;60,1) para as doses aplicadas, 45,4% (IC_95%_ 37,2;53,9) para doses válidas e de 15,5% (IC_95%_ 10,6;22,2) para as doses oportunas (Figura 2).

**Figura 2:** Cobertura completa das vacinas que deveriam ser tomadas até os 15 meses de vida (exceto contra febre amarela), doses aplicadas, doses válidas e doses oportunas, Inquérito Nacional de Cobertura Vacinal, Natal, Rio Grande do Norte, 2017-2018 (n=688)

Na análise da cobertura completa de doses aplicadas, para as vacinas que deveriam ser tomadas nos primeiros 15 meses de vida, em relação às variáveis características das crianças (Tabela 1), da mãe (Tabela 2) e da família (Tabela 3), destaca-se maior cobertura entre as crianças do sexo feminino com 53,1% (RP=1,08; IC_95%_ 0,78;1,48), de raça/cor da pele preta com 51,1% (RP=1,11; IC_95%_ 0,56;2,17) e parda com 61% (RP=1,32; IC_95%_ 0,87;2,01). 

**Tabela 1:** Análise descritiva e bivariada com razão de prevalência (RP) e intervalos de confiança de 95% (IC_95%_) da cobertura vacinal completa das vacinas a serem aplicadas até os 15 meses de vida (exceto contra febre amarela) considerando as doses aplicadas, de acordo com as características das crianças, Inquérito Nacional de Cobertura Vacinal, Natal, Rio Grande do Norte, 2017-2018 (n=688)

Variáveis	Cobertura % (IC_95_ **%**)	RP bruta (IC_95_ **%**)
**Ordem de nascimento**		
Primeiro	48,5 (36,0;61,3)	1
Segundo	52,2 (35,7;68,2)	1,07 (0,70;1,64)
Terceiro	64,0 (45,3;79,3)	1,31(0,90;1,93)
Acima do terceiro	51,7 (34,7;68,3)	1,06 (0,71;1,57)
**Sexo da criança**		
Masculino	49,0 (37,3;60,8)	1
Feminino	53,1 (40,6;65,3)	1,08 (0,78;1,48)
**Raça/cor da pele da criança**		
Branca	45,9 (33,0;59,5)	1
Preta	51,1 (23,4;77,8)	1,11 (0,56;2,17)
Parda	61,0 (47,2;73,2)	1,32 (0,87;2,01)
**Frequenta berçário/creche**		
Sim	51,7 (39,4;63,5)	1
Não	50,2 (34,2;66,2)	0,97 (0,62;1,50)

**Tabela 2:** Análise descritiva e bivariada com razão de prevalência (RP) e intervalos de confiança de 95% (IC_95%_) da cobertura vacinal completa das vacinas recomendadas para crianças com até os 15 meses de vida (exceto febre amarela), considerando doses aplicadas, de acordo com as características maternas das crianças, Inquérito de Cobertura Vacinal, Natal, Rio Grande do Norte, 2017-2018 (n=688)

Variáveis	Cobertura % (IC_95_ **%**)	RP bruta (IC_95_ **%**)
**Anos de estudo**		
≤8	50,8 (38,2;63,3)	1
9-12	76,7 (59,7;87,9)	1,51 (1,16;1,96)
13-15	55,3 (42,2;67,7)	1,08 (0,75;1,56)
≥16	45,8 (30,9;61,5)	0,90 (0,59;1,37)
**Idade da mãe (anos)**		
<20	75,7 (31,9;95,3)	1
20-34	48,6 (37,7;59,6)	0,64 (0,38;1,06)
≥35	53,1 (40,2;65,5)	0,70 (0,41;1,17)
**Raça/cor da pele da mãe**		
Branca	43,9 (27,1;62,2)	1
Preta	62,6 (47,6;75,5)	1,42 (0,88;2,29)
Parda	58,9 (45,6;71,1)	1,34 (0,76;2,34)
**Trabalho materno**		
Sim	49,1 (35,9;62,5)	1,18 (0,87;1,60)
Não	58,2 (50,2;65,7)	1
**Situação conjugal**		
Possui companheiro	55,6 (45,1;65,7)	0,84 (0,47;1,50)
Não possui companheiro	46,8 (26,0;68,8)	1

**Tabela 3:** Análise descritiva e bivariada com razão de prevalência (RP) e intervalos de confiança de 95% (IC_95%_) da cobertura vacinal completa das vacinas a serem aplicadas até os 15 meses de vida (exceto febre amarela) considerando doses aplicadas, de acordo com as características da família das crianças, Inquérito de Cobertura Vacinal, Natal, Rio Grande do Norte, 2017-2018 (n=688)

Variáveis	Cobertura % (IC_95_ **%**)	RP bruta (IC_95_ **%**)
**Aglomeração intradomiciliar**		
Sim (acima de 4 pessoas/cômodo)	51,8 (41,6;61,9)	0,70 (0,36;1,35)
Não (0 a 3 pessoas/cômodo)	36,6 (18,4;59,6)	1
**Recebe Bolsa família**		
Sim	52,0 (38,6;65,2)	1
Não	50,5 (38,9;62,0)	0,97 (0,68;1,37)
**Presença da avó no domicílio**		
Sim	56,1 (38,7;72,1)	1
Não	48,4 (35,4;61,6)	0,86 (0,54;1,37)
**Renda familiar mensal (R$)**		
<1.000	57,8 (50,7;64,6)	1
1.000-3.000	62,2 (45,7;76,4)	1,07 (0,81;1,41)
3.000-8.000	64,2 (34,7;85,8)	1,11 (0,70;1,74)
≥8.000	40,5 (8,5;83,4)	0,70 (0,21;2,31)
Não sabe/não respondeu	46,8 (23,1;72,2)	0,59 (0,33;1,06)
**Estrato socioeconômico**		
Estrato A e B	42,4 (29,6;56,2)	1
Estrato C e D	61,3 (54,7;67,4)	1,44 (1,03;2,02)
**Uso de serviço privado para vacinação**		
Não	54,0 (29,2;77,0)	1
Sim	49,7 (37,4;62,0)	0,91 (0,49;1,69)

Houve maior cobertura vacinal nas seguintes variáveis: filhos de mulheres com escolaridade entre 9 e 12 anos de estudo com 76,7% (RP=1,51; IC_95%_ 1,16;1,96), de mães com idade abaixo dos 20 anos apresentando 75,7% (RP=1; IC_95%_), de raça/cor da pele preta com 62,6% (RP=1,42; IC_95%_ 0,88;2,29), mães que não trabalham com 58,2% (RP=1; IC_95%_), possuem companheiro 55,6% (RP=0,84; IC_95%_ 0,47;1,50), recebem e receberam Bolsa Família 52% (RP=1; IC_95%_), tem a presença da avó no domicílio 56,1% (RP=1; IC_95%_), crianças inseridas no estrato socioeconômico C e D com 61,3% de cobertura (RP=1,44; IC_95%_ 1,03;2,02) e que não usam o serviço privado para a vacinação com 54% (RP=1; IC_95%_).

Na análise bivariada, houve maior cobertura vacinal para as variáveis: mães com tempo de estudo entre 9 e 12 anos de estudo (RP=1,51; IC_95%_ 1,16;1,96) (Tabela 2), com ênfase para crianças dos estratos socioeconômicos C e D (RP=1,44; IC_95%_ 1,03;2,02) (Tabela 3).

## DISCUSSÃO

Os resultados desta pesquisa apresentaram cobertura vacinal completa para crianças até os 15 meses de vida em Natal-Rio Grande do Norte abaixo das metas preconizadas pelo PNI. Os dados tornam-se mais preocupantes quando são observados os baixos percentuais de doses oportunas. 

Estudo realizado em Rondonópolis-Mato Grosso avaliou a cobertura das vacinas preconizadas no calendário vacinal para crianças de até 12 meses. Nesse estudo identificou a cobertura oportuna abaixo da meta estabelecida pelo PNI que é de 90,0%, corroborando os resultados deste estudo.^11^

O alerta para o processo de queda na cobertura vacinal no Brasil também foi descrito por Barbieri, que em 2017 identificou que somente a vacina BCG atingiu a meta preconizada pelo programa. Em 2019, todos os imunobiológicos constantes no calendário de vacinação infantil brasileiro apresentaram coberturas abaixo da meta preconizada.^12^ Tal fenômeno também é observado em outros países. Um relatório elaborado em 2019 pela OMS, pelo Fundo das Nações Unidas para a Infância e pela Estimates of National Immunization Coverage destaca que, entre os 193 Estados-membros da OMS, não houve cobertura acima de 90,0% em nenhuma das vacinas preconizadas no calendário infantil.^13,14,15^

As vacinas proporcionam a erradicação e o controle de doenças infecciosas, como a poliomielite e a varíola, e a redução da mortalidade infantil. Recentemente, evitaram a morte de mais de 20 milhões de pessoas durante a pandemia da covid-19. Apesar da comprovada e inquestionável eficácia das vacinas, observa-se uma tendência global de redução das coberturas vacinais, o que gera riscos para o ressurgimento de doenças já erradicadas e controladas.^16,17^

Estudo ecológico realizado na Paraíba sinalizava para a baixa cobertura vacinal de alguns desses imunobiológicos, em especial para BCG.^18^ A inconstância na disponibilidade de imunobiológicos nos serviços de atenção básica também pode ter influenciado a baixa cobertura. É importante ressaltar que o Ministério da Saúde publicou em 2017 nota informativa acerca do desabastecimento da vacina BCG e a vacina contra hepatite B. Isso pode ter impacto direto na vacinação oportuna, uma vez que os imunobiológicos são administrados ao nascer.^19^

A irregularidade no fornecimento e na disponibilidade dos imunizantes nos serviços de atenção primária são fatores que impactam diretamente a cobertura vacinal. Estudo publicado em 2018 analisou a tendência da disponibilidade de vacinas no Brasil em 2012, 2014 e 2018. Identificou que, apesar de haver uma tendência crescente na disponibilidade de todas as vacinas investigadas, cerca de um quarto das unidades básicas de saúde brasileiras não dispunha de todas as vacinas em 2018. O desabastecimento, mesmo que em curto prazo, fragiliza a continuidade do cuidado e pode causar prejuízos na confiabilidade do PNI.^20^

Estudo realizado por Baralhas et al.,^21^ em um município do estado de São Paulo, identificou que as famílias com melhores condições financeiras se negam a aceitar visita dos agentes comunitários de saúde e acreditam que não precisam fazer o cadastro no SUS, devido ao fato de possuírem planos de saúde. Destacando as análises realizadas neste estudo, demonstra-se o impacto socioeconômico direto no acesso aos serviços de saúde pública. 

Alguns estudos associaram a baixa escolaridade dos pais, a menor renda e a raça/cor da pele preta da mãe como fatores determinantes para a baixa imunização.^22,23^ Os resultados deste estudo confirmam uma mudança no cenário mundial, já descrito em estudos mais recentes que demonstram maior risco de incompletude do calendário vacinal em famílias de classes econômicas mais altas.^6,7^ A identificação na mudança desses fatores associados é de grande importância para que ocorra direcionamento na formulação de políticas públicas de saúde. 

Este estudo identificou que há uma menor cobertura vacinal entre crianças que possuem mães que trabalham, apesar de não haver significância estatística. Esse resultado pode estar relacionado ao horário de funcionamento das salas de vacinação. Um estudo^25^ identificou que a restrição de horários das salas de vacinação torna-se uma barreira no acesso aos serviços de saúde, visto que grande parte da população está inserida no mercado de trabalho e dispõe de menor tempo hábil para procurar tal serviço. Os autores ressaltam que algumas salas de vacinação não cumprem o horário da atenção primária à saúde. Existem salas que funcionam apenas no período da tarde, e outras, uma vez por semana, por exemplo. 

Em 2019, o Ministério da Saúde anunciou plano de medidas para combater as baixas coberturas vacinais. Os dez passos para garantir a ampliação das coberturas vacinais incluem medidas como manter as salas de vacinação abertas durante todo o horário de funcionamento da unidade, a não obrigatoriedade de apresentação do comprovante de residência no ato da vacinação, utilizar os espaços de consultas e procedimentos de outros profissionais para orientar sobre a atualização da carteira de vacinação, monitorar os usuários que estão com vacinas atrasadas e realizar busca ativa.^27^

A queda na cobertura vacinal é seguida por ampla discussão sobre a obrigatoriedade da vacina para crianças, segundo o Estatuto da Criança e do Adolescente, Lei nº 8.069/1990, art. 14, parágrafo primeiro, “é obrigatória a vacinação das crianças nos casos recomendados pelas autoridades sanitárias”.^28^ Muitos pais e responsáveis optam pela não vacinação, sendo assim alguns municípios em busca de soluções para baixa cobertura vacinal aprovaram leis que condicionam a matrícula escolar com apresentação da caderneta de vacinação atualizada. O Ceará instituiu a Lei nº 16.929/2019 que obriga a apresentação da carteira de vacinação atualizada nas instituições públicas e privadas de educação infantil ou de ensino médio.^29^ Em Natal, apesar de solicitar em edital o comprovante de vacinação no ato da matrícula, a obrigatoriedade não é condicionada. 

Entre as limitações deste estudo, destaca-se o viés de informação e de memória por parte dos participantes do estudo que também podem ter influenciado os resultados. Houve um percentual de recusa alto entre as famílias de classes econômicas A (60,4%) e B (47,5%). Ressalta-se que a análise da cobertura vacinal, com dados fidedignos, configura-se como um importante instrumento para nortear as características de uma doença na população e para estabelecer ações mais efetivas de controle de doenças potencialmente preveníveis.

No contexto da saúde coletiva, especialmente, os resultados observados permitem que os gestores de saúde a nível municipal, estadual e federal possam atuar em conjunto na logística para evitar o atraso e desabastecimento dos imunobiológicos nas salas de vacinação, assim como, planejar ações capazes de aumentar a adesão da população a vacinação infantil. Os resultados deste estudo alertam para a importância da educação em saúde dos pais e responsáveis pela criança, de forma a favorecer o conhecimento e o reconhecimento da essencialidade da vacinação. Medidas como intensificar a divulgação do calendário vacinal por meio das mídias digitais, facilitar o acesso aos serviços de saúde, com horários mais flexíveis nas salas de vacinação, formular e implantar políticas de saúde que promovam o fortalecimento da vigilância em saúde e realizar inquéritos domiciliares frequentemente podem contribuir para o aumento da cobertura vacinal em crianças com até 15 meses de vida. 

É importante ressaltar que as ações de saúde devem ser voltadas para o acesso universal de toda a população. Visa-se garantir o alcance de metas estabelecidas, importantes na prevenção e erradicação de doenças preveníveis, e consequentemente evitar o ressurgimento de doenças erradicadas que assolaram o mundo em séculos passados.
